# Erythropoietin Dose and Mortality in Hemodialysis Patients: Marginal Structural Model to Examine Causality

**DOI:** 10.1155/2016/6087134

**Published:** 2016-05-19

**Authors:** Elani Streja, Jongha Park, Ting-Yan Chan, Janet Lee, Melissa Soohoo, Connie M. Rhee, Onyebuchi A. Arah, Kamyar Kalantar-Zadeh

**Affiliations:** ^1^Harold Simmons Center for Kidney Disease Research and Epidemiology, School of Medicine, University of California, Irvine, Orange, CA, USA; ^2^Division of Nephrology, Ulsan University Hospital, University of Ulsan College of Medicine, Ulsan, Republic of Korea; ^3^Department of Epidemiology, UCLA Fielding School of Public Health, Los Angeles, CA, USA; ^4^Division of Nephrology and Hypertension, School of Medicine, University of California, Irvine, Orange, CA, USA

## Abstract

It has been previously reported that a higher erythropoiesis stimulating agent (ESA) dose in hemodialysis patients is associated with adverse outcomes including mortality; however the causal relationship between ESA and mortality is still hotly debated. We hypothesize ESA dose indeed exhibits a direct linear relationship with mortality in models of association implementing the use of a marginal structural model (MSM), which controls for time-varying confounding and examines causality in the ESA dose-mortality relationship. We conducted a retrospective cohort study of 128 598 adult hemodialysis patients over a 5-year follow-up period to evaluate the association between weekly ESA (epoetin-*α*) dose and mortality risk. A MSM was used to account for baseline and time-varying covariates especially laboratory measures including hemoglobin level and markers of malnutrition-inflammation status. There was a dose-dependent positive association between weekly epoetin-*α* doses ≥18 000 U/week and mortality risk. Compared to ESA dose of <6 000 U/week, adjusted odds ratios (95% confidence interval) were 1.02 (0.94–1.10), 1.08 (1.00–1.18), 1.17 (1.06–1.28), 1.27 (1.15–1.41), and 1.52 (1.37–1.69) for ESA dose of 6 000 to <12 000, 12 000 to <18 000, 18 000 to <24 000, 24 000 to <30 000, and ≥30 000 U/week, respectively. High ESA dose may be causally associated with excessive mortality, which is supportive of guidelines which advocate for conservative management of ESA dosing regimen in hemodialysis patients.

## 1. Introduction

Over the past 20 years, erythropoiesis stimulating agents (ESAs) have been a mainstay in anemia treatment in chronic kidney disease (CKD) and end-stage renal disease (ESRD) patients. Although anemia treatment improves survival in this population, correction of hemoglobin (Hb) to a normal range using ESAs did not demonstrate an additional benefit in previous randomized trials [1–4]. Furthermore, two of these trials unexpectedly showed worse outcomes in patients randomized to achieve higher Hb targets above 13 g/dL [3, 4]. In those studies, the mean ESA dose was greater in the high Hb target arm than in the lower Hb target arm. It has been debated whether high ESA dose mediates the excess observed mortality risk.

As ESA dose and other markers of nutrition and inflammation change over time, the ESA-mortality association remains vulnerable to biases that would arise using conventional survival models [5]. A marginal structural model (MSM) is a type of analysis which can address time-varying covariates that may simultaneously act as a confounder and intermediate variable [6–8]. Notably, Hb level fits this description as it is a critical time-varying confounder that is affected by the previous ESA dose, and it influences the future ESA dose and survival, upon evaluating ESA dose-mortality associations. Furthermore, time-varying ESA dose and nutritional markers may be associated with a greater likelihood of informed censoring. The MSM method attempts to account for these potential time-varying biases by creating weights for each patient at each time interval. These weights estimate the inverse probability of a patient being at his or her exposure (ESA) level for that time interval, and them not having been censored at a prior time interval. The weights are constructed according to baseline and time-varying covariates and attempts to address time-varying confounding leading to ESA dose fluctuations (changes in exposure level) or informative censoring (ESA dose leading to a higher probability of kidney transplant). Holding particular assumptions true in the use of MSM, associations found from MSM are believed to have a causal interpretation. Thus using a MSM and a large cohort of hemodialysis (HD) patients, we aimed to examine the causal effects of weekly epoetin-*α* dose levels and mortality.

## 2. Materials and Methods

### 2.1. Study Cohort

**Figure 1:**
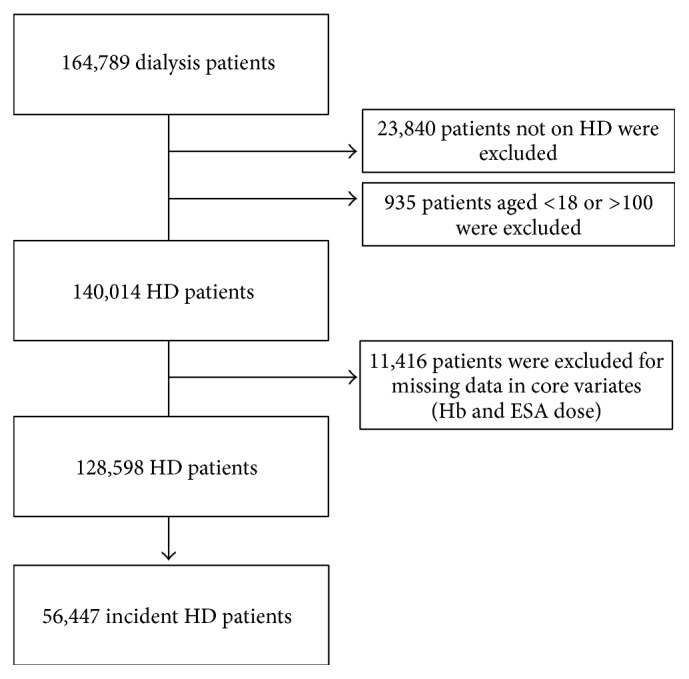
Flowchart of patient selection.

Among a total of 164 789 ESRD patients receiving dialysis treatment from July 1, 2001, through June 30, 2006, in any one of the outpatient facilities of a large dialysis organization (DaVita Healthcare Partners) in the United States, we examined data from 128 598 patients who met the following inclusion criteria: being of age ≥18 years, having underwent HD for at least 90 days, and having had complete data on the main exposure and core covariate (ESA dose and Hb level) (Figure 1). In sensitivity analyses, we restricted analyses to an incident HD cohort, defined as patients whose HD duration at cohort entry was less than 6 months. The institutional review committees of Los Angeles Biomedical Research Institute at Harbor-UCLA, University of California, Irvine, and DaVita Clinical Research approved this study. Given the large sample size, anonymity of the patients studied and nonintrusive nature of the research, the requirement for consent was waived.

### 2.2. Dose of Erythropoiesis Stimulating Agent

The primary exposure was weekly epoetin-*α* dose (U/week), which was calculated and averaged for every 3-month interval (calendar quarter) in order to minimize measurement variability. ESA dose was divided into 6 preselected ordinal categories: <6 000 U/week (reference), 6 000 to <12 000 U/week, 12 000 to <18 000 U/week, 18 000 to <24 000 U/week, 24 000 to <30 000 U/week, and ≥30 000 U/week. ESA <6 000 U/week was designated as the reference group. ESA dose during hospitalization was not available in this cohort. The in-hospital ESA dose was imputed using the most recent ESA dose prior to hospitalization.

### 2.3. Study Outcomes

The primary outcome was all-cause mortality, and the secondary outcomes were cardiovascular (CV) or infectious mortality (see Supplement Table 1 in Supplementary Materials available online at http://dx.doi.org/10.1155/2016/6087134). Detailed information on cause of death was obtained from the US Renal Data System (USRDS) “CDeath” codes, which are derived from the ESRD Death Notification Form (CMS-2746) provided by ESRD networks to the USRDS. Cause of death was categorized as cardiovascular, infectious, or others by clinician decision according to these “CDeath” codes. Patients were followed until the time of death or the end of study period (June 30, 2007). Patients were censored at the time of renal transplantation, change of dialysis modality, that is, HD to peritoneal dialysis, or transfer to a non-DaVita facility.

### 2.4. Covariates of Interest

Demographic covariates included baseline age, sex, race/ethnicity (Caucasian, African American, Hispanic, Asian, and others), marital status (married, divorced, single, and widowed), primary insurance (Medicare, Medicaid, private insurance, and others), comorbid conditions (see below), calendar quarter of cohort entry, and dialysis vintage (<6 months, 6 months to <24 months, 2 to <5 years, and ≥5 years), for which information was obtained from the USRDS. The following comorbidities were considered: diabetes mellitus, hypertension, ischemic heart disease, congestive heart failure, cerebrovascular disease, peripheral vascular disease, chronic obstructive pulmonary disease, malignancy, nonambulatory state, and current smoking status. Dialysis duration was defined as the duration of time between the first day of dialysis treatment and the first day that patients entered the cohort.

Time-varying lab covariates, also averaged over a successive 3-month (calendar quarter) interval, included Hb level, serum albumin, creatinine, calcium, phosphorus, bicarbonate, total iron binding capacity, ferritin, white blood cell count, lymphocyte percentage, normalized protein nitrogen appearance (a metric of dietary protein intake), dialysis adequacy (single-pool *Kt*/*V*), and body mass index. Hb level was measured approximately twice per month. Most laboratory data were measured monthly, except for serum ferritin level that was measured at least quarterly. Blood samples were drawn before HD using uniform techniques in all dialysis clinics and were transported to the central laboratory, usually within 24 hours (DaVita Laboratory, Deland, FL). All laboratory values were measured via automated and standardized methods. Post-HD body weight was used to calculate body mass index.

### 2.5. Statistical Analysis

Inverse probabilities of treatment weights (IPTWs) were created on the basis of the inverse of the predicted probability of a patient receiving the treatment that was actually received (the above-mentioned ESA dose categories), given the baseline and time-varying covariates. We used ordinal logistic regression to calculate IPTWs at baseline and for each subsequent quarter of follow-up [9]. IPTWs can result in excessively large weights when there is an atypical treatment decision or data error. Hence, stabilized IPTWs have been applied to reduce the potential for extreme IPTWs. In the stabilized IPTWs, the numerator is the calculated probability of the observed treatment (ESA dose) given the previous ESA dose and baseline patient characteristics, while the denominator is the calculated probability of ESA dose, previous ESA dose, and both baseline and time-varying covariates. Baseline covariates included age, gender, race, insurance, marital status, comorbidities, and baseline lab values, while time-varying covariates included time-updated quarterly lab values and their respective lag (previous quarter) values. Estimated weights were then truncated at the 1st and 99th percentile values and used in the analyses [10].

To address informative censoring, we fitted logistic regression models to calculate the inverse probability of censoring weights (IPCWs) at each time interval. As done with IPTWs, we used the same covariates for the numerator and denominator of the stabilized IPCW modeling the calculated probability of observed censorship. As large censoring weights were not observed, truncation was not performed for IPCW values. The final stabilized weights were calculated as the product of the stabilized IPTWs and stabilized IPCWs.

We estimated the odds ratio (OR) using a generalized estimating equation that included ESA dose category and the final stabilized weights on the basis of all baseline covariates. The ESA dose category of <6 000 U/week was treated as the reference group. Missing values for baseline covariates were imputed using multiple imputation with 5 iterations. In both the overall and incident study populations, data were missing for less than 5% and 1%, respectively. To further impute missing time-varying covariates in each time window, we used last-value carried forward. All analyses were conducted using SAS version 9.3 (SAS Institute Inc., Cary, NC).

## 3. Results

### 3.1. Patient's Characteristics

Baseline characteristics of the overall patient cohort and stratified across ESA categories are summarized in Table 1. During the baseline quarter, there were 6 644 (5%), 23 314 (18%), 26 852 (21%), 21 487 (17%), 15 278 (12%), and 35 023 (27%) patients receiving a weekly ESA dose of <6 000, 6 000 to <12 000, 12 000 to <18 000, 18 000 to <24 000, 24 000 to <30 000, and ≥30 000 U/week. The mean ± standard deviation (SD) age was 62 ± 15 years, 55% of the patients were women, 32% and 14% were African American and Hispanic, respectively, and 57% were diabetic. The baseline mean ± SD Hb level was 12.1 ± 1.0 g/dL, and the median (interquartile range, IQR) duration of follow-up was 2.2 (1.2–3.6) years. Patients receiving a higher ESA dose tended to be African American and male and had lower albumin and lymphocyte percentage.

### 3.2. Distribution of Weights

The distribution of the weights is displayed in Table 2. Stabilized weights had a maximum value of 80.9 and 78.9 in the overall and incident patient cohorts, respectively. The mean stabilized weights were 0.84 and 0.88, respectively.

### 3.3. Weekly ESA Dose and All-Cause Mortality

**Figure 2:**
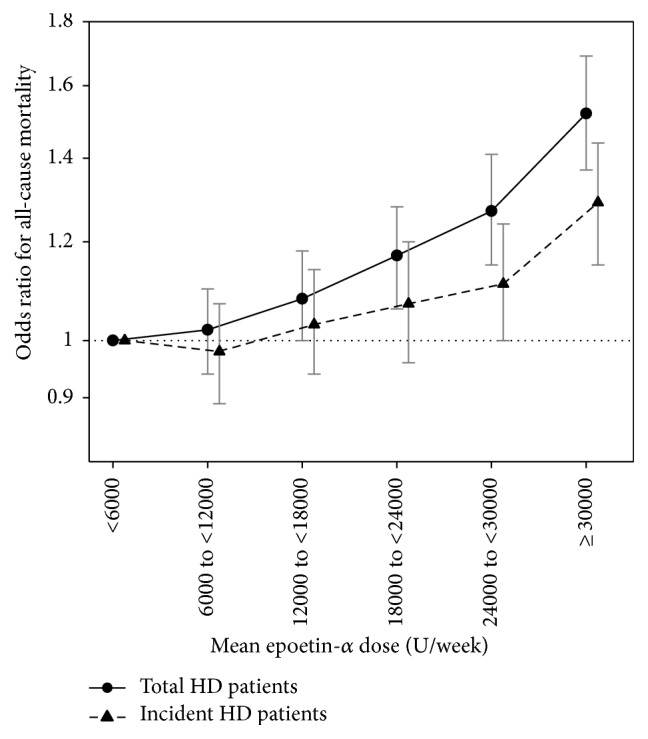
Adjusted mortality risk for all-cause mortality by weekly epoetin-*α* dose estimated by marginal structural model.

Weekly ESA doses ≥18 000 U/week were associated with higher risks of death as compared with a weekly epoetin-*α* dose of <6 000 U/week. Furthermore, a dose-response relationship was also observed (Figure 2). Weekly ESA doses of 18 000 to <24 000, 24 000 to <30 000, and ≥30 000 U/week showed 17%, 27%, and 52% higher risk of mortality, respectively (Table 3).

### 3.4. Weekly ESA Dose and Cardiovascular/Infectious Mortality

Weekly ESA dose also showed a strong relationship with CV mortality risk (Table 3). Weekly ESA doses of 6 000 to <12 000, 12 000 to <18 000, 18 000 to <24 000, 24 000 to <30 000, and ≥30 000 U/week showed 13%, 21%, 23%, 35%, and 44% higher risks of CV mortality, respectively. Risk of infectious death was only significantly increased in weekly ESA dose of ≥30 000 U/week (Table 3), while ESA levels <30,000 U/week exhibited a trend toward higher risk of infectious death, compared to reference.

### 3.5. Sensitivity Analyses

In incident patients (*n* = 56 447), the relationship between weekly ESA dose and mortality risk was less apparent compared to the overall cohort, which included prevalent patients (Figure 2 and Table 4). Only a weekly ESA dose ≥30 000 U/week was significantly associated with higher mortality compared to a weekly ESA dose of <6 000 U/week (OR: 1.29, 95% confidence interval (CI): 1.15–1.44).

**Figure 3:**
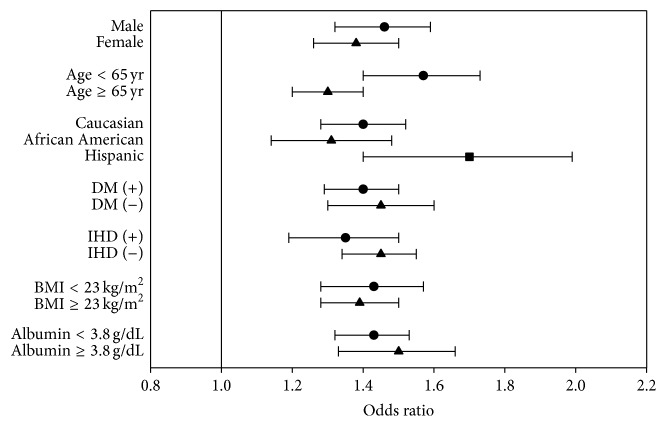
Adjusted mortality risk of weekly epoetin-*α* dose ≥30 000 versus <30 000 U/week (reference) among various subgroups. DM = diabetes mellitus, IHD = ischemic heart disease, and BMI = body mass index.

Based on our observation that a weekly ESA dose of ≥30 000 U/week was associated with a higher risk of mortality in both the overall and incident patient cohorts, we dichotomized weekly ESA dose with cutoffs at 30 000 U/week. Also, using a MSM, we then reexamined mortality risk of weekly ESA doses ≥30 000 versus <30 000 U/week (reference) in various subgroups: men versus women, age ≥65 versus <65 years, race/ethnicity (Caucasian versus African American versus Hispanic), diabetic versus nondiabetic, prior history versus no history of ischemic heart disease, body mass index ≥23 versus <23 kg/m^2^, and serum albumin level ≥3.8 versus <3.8 g/dL. Among all subgroups, adjusted ORs were significantly higher in weekly ESA dose of ≥30 000 U/week than in that of <30 000 U/week (Figure 3).

## 4. Discussion

In this study, we used a MSM to evaluate the relationship between ESA dose and mortality in a large cohort of HD patients. We observed a dose-dependent relationship as higher ESA dose was associated with a higher risk of mortality.

After recent randomized trials showed worse outcomes in patients randomized to higher Hb targets [3, 4], the US Food and Drug Administration recommended a more conservative ESA dosing regimen for the treatment of patients with CKD [11]. However, the causal relationship between ESA dose and mortality has still been debated and the ideal ESA dosing regimen remains unknown. The examination of the causal effect of ESA is challenging due to the strong relationship between patient's comorbidity and ESA requirements and especially with the presence of time-dependent confounding of Hb in observational studies. The current ESA dose is influenced by previous ESA dose and Hb and affects future ESA dose and Hb. In addition, Hb itself may affect patient's outcome simultaneously. However, an increase in Hb independent of ESA dose may not be associated with higher mortality risk [12]. In presence of this type of complex confounding, traditional survival models are limited in their capacity to estimate unbiased exposure effect [5]. We used a MSM to control this time-dependent confounding [6, 7, 10, 13]. We observed significantly higher OR estimates in ESA doses over 18 000 U/week as compared to that of <6 000 U/week. Weekly ESA dose ≥30 000 U/week showed a 52% increased mortality risk.

Previous studies have tried to control time-dependent confounding using MSM, but they have reported conflicting results. Zhang et al. reported no harmful effect of median cumulative ESA dose over 30 000 U/week in elderly (≥65 years old) HD patients [14]. However, an additional study by the same group investigating a larger cohort later reported a 32% increased risk of mortality with ESA dose greater than 40 000 U/week compared with 20 000 to 30 000 U/week especially in diabetic elderly patients [15]. Wang et al. reported a mortality hazard ratio for the highest ESA doses (>49 000 U/2 weeks) of 0.98 (95% CI: 0.76–1.74) compared with ESA dose ≤14 000 U/2 weeks. They concluded that there is appreciable confounding by indication at higher ESA doses and that ESA dose was not associated with increased mortality in analysis using MSM [9]. Recently, in a European cohort, Suttorp et al. reported that the excess mortality risk for patients with high ESA dose did not fully disappear within analysis using the MSM approach. The MSM estimated a hazard ratio of 1.54 (95% CI: 1.08–2.18) for patients with ESA dose above 6 000 U/week compared to the counterpart of less than 6 000 U/week, which is in line with our results [16].

Our results should be interpreted carefully. The MSM estimates what would happen if a patient is always exposed to higher doses of ESA, which is difficult to interpret in clinical practice but nonetheless begs the question if a patient treated with a lower dose of ESA would benefit more from treatment than with a higher ESA dose. In addition, our results could be interpreted causally under the fundamental assumptions of MSM. An important assumption is “positivity,” the condition that there are both exposed and unexposed individuals at every level of the confounders. A relatively large study may have zero proportion for particular exposure and covariate histories as the number of covariates increases. Estimated weights with a mean far from one or very extreme values are indicative of nonpositivity or misspecification of the weight model. In our study, it could be debated whether the mean of the stabilized weights of 0.84 in overall patients and 0.88 in incident patients is close enough to one to justify the conclusion that models were well specified. However, there is a tradeoff between reducing confounding bias and increasing bias/variance due to nonpositivity [10]. The distribution of weights in our study is comparable to that of other MSM studies although the mean of weights in our study is smaller [9, 16].

Thin evidence for biologic plausibility also dampens a conclusive consensus of a causal relationship between ESA dose and mortality. Considering that the most common cause of death in CKD patients is related to CV disease, a harmful effect of high ESA dose, if any, would likely be mediated by an adverse effect due to CV disease. The treatment with exogenous erythropoietin is distinctly different from the normal biology. There is a very rapid rise and supraphysiologic peak in serum concentration of erythropoietin after injection, followed by a rapid decline [17, 18]. Repetitive supraphysiologic stimulation could disorder cardiac modeling, increase vulnerability to stress, or impair the ability of higher Hb to diminish left ventricular hypertrophy [18–20]. Another hypothesis is that iron depletion, increased platelet reactivity and platelet numbers [21], and associated relative thrombocytosis might contribute to increased CV events upon administering a high ESA dose [22]. Moreover, high ESA dose may directly lead to thrombocytosis [23]. A 2014 review summarized additional potential mechanisms, including ESA effect on arterial blood pressure via increasing blood viscosity and vasoconstriction [24]. In our study, estimated ORs for CV death were greater than those for all-cause mortality, although the differences were small (Tables 3 and 4). Further investigations are warranted to validate biologic plausibility.

The association between ESA dose and mortality was observed even in incident patients, but the strength of association was weaker than in the overall cohort, which additionally included prevalent patients. This may be due to the relatively small number of patients or the cumulative effect of ESA exposure. Prevalent patients receiving a high ESA dose during the study period tend to have greater cumulative exposure to ESA prior to enrollment. This observation may not be present in incident HD patients because ESA dose administered during the predialysis CKD stages is usually lower compared to the dose administered during maintenance HD. If a cumulative effect of ESA exposure exists, estimated ORs may be augmented in the overall cohort compared to that of the incident cohort [25]. Another speculation is that, in the early period after HD initiation, other mortality risk factors are more dominant than ESA dose. Survivor bias is more likely to affect studies of prevalent patients opposed to incident patients.

Our study has several limitations. First, the validity of our analysis depends on the assumption that we have adjusted for all confounders (exchangeability). Given the detailed level of information on a variety of demographics, laboratory parameters, and dialysis adequacy in our data, we believe that we have controlled for the most important confounding. However, the possibility of residual confounding or confounding by indication cannot be completely excluded. Although we included serum albumin, white blood cell counts, and lymphocyte percentage in the models [26], we did not have data for other inflammatory markers, such as C-reactive protein [27, 28]. It might be interpreted as a high OR estimate for infectious mortality with ESA dose over 30 000 U/week in our results. Considering that there is no evidence to date that ESA causes infectious complications, a small effect on infectious mortality could indicate residual confounding. Second, the estimates for our MSM could be affected by the level of weight truncation, which is reflective of the tradeoff between control of confounding and precision of our effect estimates [10]. Third, our data did not contain information on ESA dose during hospitalization. We imputed ESA doses assuming thrice-weekly dosing using the prehospitalization dose. Although this approach may not reflect actual in-hospital ESA dosing exactly, it may be an alternative solution to address this missing ESA data problem. Despite these limitations, our cohort is one of the largest ones to investigate the ESA-mortality association and is nationally representative of the United States adult HD population. Furthermore, our cohort was followed up for an extended period of time, giving sufficient power in this analysis.

In conclusion, estimating the causal relationship between ESA dose and mortality is complex due to the strong relationship between comorbidity, ESA requirements, and time-dependent confounding of Hb. Using a MSM, we observed a possible causal relationship between higher ESA dose and excess mortality risk in HD patients. It supports the current conservative ESA dosing regimen which balances the benefit of anemia correction and a potential harm of higher ESA dose. Further studies (including biological and prospective studies) are warranted to establish the ideal ESA dosing algorithm in CKD and ESRD patients and to further unveil the complex pathophysiological relationship between ESA dose and mortality.

## Supplementary Material

Detailed information on cause of death was obtained from the US Renal Data System (USRDS) “CDeath” codes, which are derived from the ESRD Death Notification Form (CMS-2746) provided by ESRD networks to the USRDS. Cause of death was categorized as cardiovascular, infectious, or others by clinician decision according to these “CDeath” codes.

## Figures and Tables

**Table 1:** Patient characteristics by weekly epoetin-*α* dose categories.

	Total	Averaged weekly epoetin-*α* dose during follow-up period (U/week)					
		<6 000	6 000 to <12 000	12 000 to <18 000	18 000 to <24 000	24 000 to <30 000	≥30 000
*n* (%)	128 598 (100)	6 644 (5)	23 314 (18)	26 852 (21)	21 487 (17)	15 278 (12)	35 023 (27)
Age (yr)	62 ± 15	62 ± 16	63 ± 15	62 ± 15	62 ± 15	62 ± 15	60 ± 15
Female (%)	70 309 (55)	4 191 (63)	13 107 (56)	14 604 (54)	11 541 (54)	8 153 (53)	18 713 (53)
Dialysis vintage (mo)	3 (1,28)	16 (2,39)	7 (1,30)	4 (1,27)	2 (1,26)	2, (1,25)	2 (1,26)
Race (%)							
Caucasian	55 107 (43)	3 181 (48)	10 057 (43)	11 026 (41)	9 072 (42)	6 460 (42)	15 311 (44)
African American	41 257 (32)	1 678 (25)	6 286 (27)	8 104 (30)	6 775 (32)	5 173 (34)	13 241 (38)
Hispanic	18 409 (14)	968 (15)	4 032 (17)	4 384 (16)	3 336 (16)	2 116 (14)	3 573 (10)
Asian	3 887 (3)	218 (3)	885 (4)	1 002 (4)	675 (3)	436 (3)	671 (2)
Other	9 938 (8)	599 (9)	2 054 (9)	2 336 (9)	1 629 (8)	1 093 (7)	2 227 (6)
Insurance (%)							
Medicare	81 230 (63)	4 357 (66)	14 881 (64)	17 062 (64)	13 497 (63)	9 645 (63)	21 788 (62)
Medicaid	6 777 (5)	194 (3)	989 (4)	1 358 (5)	1 225 (6)	897 (6)	2 114 (6)
Private	12 156 (9)	623 (9)	2 344 (10)	2 718 (10)	2 138 (10)	1 414 (9)	2 919 (8)
Other	28 435 (22)	1 470 (22)	5 100 (22)	5 714 (21)	4 627 (22)	3 322 (22)	8 202 (23)
Marital status (%)							
Married	51 385 (40)	3 057 (46)	9 942 (43)	10 888 (41)	8 368 (39)	5 934 (39)	13 196 (38)
Divorced	8 725 (7)	434 (7)	1 462 (6)	1 778 (7)	1 470 (7)	1 055 (7)	2 526 (7)
Single	29 615 (23)	1 482 (22)	4 939 (21)	5 701 (21)	4 873 (23)	3 536 (23)	9 084 (26)
Widowed	16 521 (13)	823 (12)	3 186 (14)	3 641 (14)	2 783 (13)	1 990 (13)	4 098 (12)
Comorbidities (%)							
DM	73 847 (57)	3 353 (50)	12 972 (56)	15 550 (58)	12 787 (60)	9 147 (60)	20 038 (57)
HTN	96 852 (79)	4 986 (81)	17 714 (80)	20 485 (80)	16 337 (80)	11 560 (80)	25 770 (78)
IHD	26 062 (21)	1 287 (21)	4 777 (22)	5 549 (22)	4 470 (22)	3 192 (22)	6 787 (20)
CHF	33 534 (28)	1 475 (24)	5 663 (26)	7 005 (27)	5 691 (28)	4 249 (29)	9 451 (28)
PVD	13 820 (11)	669 (11)	2 404 (11)	2 842 (11)	2 373 (12)	1 770 (12)	3 762 (11)
CVA	9 015 (7)	482 (8)	1 628 (7)	1 928 (8)	1 632 (8)	1 025 (7)	2 320 (7)
COPD	6 904 (6)	377 (6)	1 140 (5)	1 365 (5)	1 135 (6)	887 (6)	2 000 (6)
Malignancy	5 538 (5)	209 (3)	829 (4)	1 019 (4)	879 (4)	622 (4)	1 980 (6)
Current smoking	5 854 (5)	345 (6)	978 (4)	1 134 (4)	971 (5)	650 (4)	1 776 (5)
BMI (kg/m^2^)	26.6 ± 6.7	26.7 ± 6.5	26.2 ± 6.0	26.4 ± 6.4	26.7 ± 6.5	26.8 ± 6.9	27.1 ± 7.4
Laboratory parameters							
Hemoglobin (g/dL)	12.1 ± 1.0	12.6 ± 0.8	12.4 ± 0.7	12.3 ± 0.8	12.2 ± 0.8	12.0 ± 0.9	11.5 ± 1.2
Creatinine (mg/dL)	8.2 ± 3.0	8.4 ± 3.2	8.4 ± 3.0	8.4 ± 3.0	8.3 ± 3.0	8.1 ± 3.0	8.0 ± 3.1
Albumin (g/dL)	3.7 ± 0.4	3.9 ± 0.3	3.9 ± 0.3	3.8 ± 0.4	3.7 ± 0.4	3.7 ± 0.4	3.5 ± 0.5
TIBC (mg/dL)	203 ± 41	215 ± 36	211 ± 36	206 ± 37	203 ± 39	201 ± 40	194 ± 46
Calcium (mg/dL)	9.3 ± 0.6	9.4 ± 0.6	9.4 ± 0.6	9.3 ± 0.6	9.3 ± 0.6	9.2 ± 0.6	9.1 ± 0.7
Phosphorus (mg/dL)	5.6 ± 1.3	5.4 ± 1.2	5.4 ± 1.1	5.5 ± 1.2	5.6 ± 1.3	5.6 ± 1.3	5.7 ± 1.4
WBC (×10^3^/mm^3^)	7.4 ± 2.4	7.4 ± 2.2	7.4 ± 2.0	7.4 ± 2.1	7.4 ± 2.2	7.5 ± 2.4	7.5 ± 2.9
Lymphocyte (%)	20.3 ± 7.3	22.3 ± 7.4	21.7 ± 7.2	21.0 ± 7.2	20.3 ± 7.2	19.6 ± 7.2	18.8 ± 7.4
Ferritin (ng/mL)	515 (314,742)	572 (377,777)	554 (362,755)	532 (341,741)	509 (313,728)	491 (291,730)	473 (265,737)
Single-pool *Kt*/*V*	1.6 ± 0.3	1.7 ± 0.3	1.7 ± 0.3	1.6 ± 0.3	1.6 ± 0.3	1.6 ± 0.3	1.5 ± 0.3
nPNA (g/kg/day)	1.0 ± 0.2	1.0 ± 0.2	1.0 ± 0.2	1.0 ± 0.2	1.0 ± 0.2	1.0 ± 0.2	0.9 ± 0.2

Note: categorical variables are expressed as frequency (percentage). Continuous variables are given as mean ± SD or median (interquartile range) as appropriate.

Conversion factors for units: hemoglobin and albumin in g/dL to g/L ×10; creatinine in mg/dL to *μ*mol/L ×88.4; calcium in mg/dL to mmol/L ×0.2495; and phosphorus in mg/dL to mmol/L ×0.3229. No conversion necessary for ferritin in ng/mL and *μ*g/L and WBC count in 10^3^/*μ*L and 10^9^/L. DM = diabetes mellitus, HTN = hypertension, IHD = ischemic heart disease, CHF = congestive heart failure, PVD = peripheral vascular disease, CVA = cerebrovascular accident, COPD = chronic obstructive pulmonary disease, BMI = body mass index, TIBC = total iron binding capacity, WBC = white blood cell, and nPNA = normalized protein nitrogen appearance.

**Table 2:** Weight distribution for marginal structural model across 3-month time intervals.

Percentile	Overall (prevalent + incident) patients			Incident patients		
	Stabilized IPTW	Stabilized IPCW	Stabilized weight	Stabilized IPTW	Stabilized IPCW	Stabilized weight
Maximum	82.6	3.66	80.9	83.9	3.74	78.7
99th	6.55	1.31	6.28	6.26	1.27	6.10
95th	2.24	1.02	2.17	2.23	1.01	2.18
90th	1.43	1.00	1.40	1.43	1.00	1.41
75th	0.92	0.99	0.92	0.94	0.99	0.93
50th (Median)	0.68	0.97	0.67	0.75	0.98	0.74
25th	0.31	0.94	0.29	0.39	0.96	0.38
10th	0.11	0.90	0.10	0.16	0.93	0.15
5th	0.05	0.86	0.05	0.09	0.90	0.08
1st	0.01	0.78	0.01	0.03	0.83	0.03
Minimum	0.0003	0.076	0.0003	0.0008	0.22	0.0008
Mean	0.86	0.97	0.84	0.90	0.98	0.88

IPTW = inverse probability of treatment weight. IPCW = inverse probability of censoring weight.

**Table 3:** Adjusted odds ratios (95% confidence interval) for mortality by weekly epoetin-*α* doses in overall patient cohort.

Epoetin-*α* (U/wk)	All-cause	Cardiovascular	Infectious
<6 000	Reference	Reference	Reference
6 000 to <12 000	1.02 (0.94–1.10)	**1.13 (1.05–1.23)**	1.12 (1.00–1.25)
12 000 to <18 000	1.08 (1.00–1.18)	**1.21 (1.10–1.32)**	1.11 (0.98–1.26)
18 000 to <24 000	**1.17 (1.06–1.28)**	**1.23 (1.12–1.36)**	1.14 (1.00–1.30)
24 000 to <30 000	**1.27 (1.15–1.41)**	**1.35 (1.22–1.50)**	1.13 (0.99–1.29)
≥30 000	**1.52 (1.37–1.69)**	**1.44 (1.29–1.59)**	**1.28 (1.11–1.48)**

Note: bold font indicates statistically significant odds ratios.

**Table 4:** Adjusted odds ratio (95% confidence interval) for mortality by weekly epoetin-*α* doses in incident patients.

Epoetin-*α* dose (U/wk)	All-cause	Cardiovascular	Infectious
<6 000	Reference	Reference	Reference
6 000 to <12 000	0.98 (0.89–1.07)	1.06 (0.96–1.18)	1.08 (0.93–1.25)
12 000 to <18 000	1.03 (0.94–1.14)	1.07 (0.95–1.19)	1.10 (0.93–1.29)
18 000 to <24 000	1.07 (0.96–1.20)	**1.14 (1.01–1.30)**	1.18 (0.99–1.40)
24 000 to <30 000	1.11 (1.00–1.24)	**1.16 (1.02–1.31)**	1.11 (0.93–1.33)
≥30 000	**1.29 (1.15–1.44)**	**1.23 (1.09–1.40)**	1.18 (1.00–1.40)

Note: incident patient was defined as having a dialysis vintage of less than 6 months at cohort entry. Bold font indicates statistically significant odds ratios.

## References

[1] Besarab A., Bolton W. K., Browne J. K. (1998). The effects of normal as compared with low hematocrit values in patients with cardiac disease who are receiving hemodialysis and epoetin. *The New England Journal of Medicine*.

[2] Drüeke T. B., Locatelli F., Clyne N. (2006). Normalization of hemoglobin level in patients with chronic kidney disease and anemia. *The New England Journal of Medicine*.

[3] Singh A. K., Szczech L., Tang K. L. (2006). Correction of anemia with epoetin alfa in chronic kidney disease. *The New England Journal of Medicine*.

[4] Pfeffer M. A., Burdmann E. A., Chen C.-Y. (2009). A trial of darbepoetin alfa in type 2 diabetes and chronic kidney disease. *The New England Journal of Medicine*.

[5] Bradbury B. D., Brookhart M. A., Winkelmayer W. C. (2009). Evolving statistical methods to facilitate evaluation of the causal association between erythropoiesis-stimulating agent dose and mortality in nonexperimental research: strengths and limitations. *American Journal of Kidney Diseases*.

[6] Robins J. M., Hernán M. Á., Brumback B. (2000). Marginal structural models and causal inference in epidemiology. *Epidemiology*.

[7] Petersen M. L., Deeks S. G., Martin J. N., van der Laan M. J. (2007). History-adjusted marginal structural models for estimating time-varying effect modification. *American Journal of Epidemiology*.

[8] Hernán M. A., Robins J. M. (2006). Estimating causal effects from epidemiological data. *Journal of Epidemiology and Community Health*.

[9] Wang O., Kilpatrick R. D., Critchlow C. W. (2010). Relationship between epoetin alfa dose and mortality: findings from a marginal structural model. *Clinical Journal of the American Society of Nephrology*.

[10] Cole S. R., Hernán M. A. (2008). Constructing inverse probability weights for marginal structural models. *American Journal of Epidemiology*.

[11] FDA Drug safty communication: modified dosing recommendations to improve the safe use of erythropoiesis-stimulating agents (ESAs) in chronic kidney disease. http://www.fda.gov/Drugs/DrugSafety/ucm259639.htm.

[12] Shah A., Molnar M. Z., Lukowsky L. R., Zaritsky J. J., Kovesdy C. P., Kalantar-Zadeh K. (2012). Hemoglobin level and survival in hemodialysis patients with polycystic kidney disease and the role of administered erythropoietin. *American Journal of Hematology*.

[13] Hernán M. Á., Brumback B., Robins J. M. (2000). Marginal structural models to estimate the causal effect of zidovudine on the survival of HIV-positive men. *Epidemiology*.

[14] Zhang Y., Thamer M., Cotter D. J., Kaufman J., Hernán M. A. (2009). Estimated effect of epoetin dosage on survival among elderly hemodialysis patients in the United States. *Clinical Journal of the American Society of Nephrology*.

[15] Zhang Y., Thamer M., Kaufman J. S., Cotter D. J., Hernán M. A. (2011). High doses of epoetin do not lower mortality and cardiovascular risk among elderly hemodialysis patients with diabetes. *Kidney International*.

[16] Suttorp M. M., Hoekstra T., Mittelman M. (2015). Treatment with high dose of erythropoiesis-stimulating agents and mortality: analysis with a sequential Cox approach and a marginal structural model. *Pharmacoepidemiology and Drug Safety*.

[17] Erslev A. J. (1991). Erythropoietin. *The New England Journal of Medicine*.

[18] Fishbane S., Besarab A. (2007). Mechanism of increased mortality risk with erythropoietin treatment to higher hemoglobin targets. *Clinical Journal of the American Society of Nephrology*.

[19] Wu H., Lee S. H., Gao J., Liu X., Iruela-Arispe M. L. (1999). Inactivation of erythropoietin leads to defects in cardiac morphogenesis. *Development*.

[20] Anagnostou A., Liu Z., Steiner M. (1994). Erythropoietin receptor mRNA expression in human endothelial cells. *Proceedings of the National Academy of Sciences of the United States of America*.

[21] Stohlawetz P. J., Dzirlo L., Hergovich N. (2000). Effects of erythropoietin on platelet reactivity and thrombopoiesis in humans. *Blood*.

[22] Streja E., Kovesdy C. P., Greenland S. (2008). Erythropoietin, iron depletion, and relative thrombocytosis: a possible explanation for hemoglobin-survival paradox in hemodialysis. *American Journal of Kidney Diseases*.

[23] Vaziri N. D. (2009). Thrombocytosis in EPO-treated dialysis patients may be mediated by EPO rather than iron deficiency. *American Journal of Kidney Diseases*.

[24] Lund A., Lundby C., Olsen N. V. (2014). High-dose erythropoietin for tissue protection. *European Journal of Clinical Investigation*.

[25] Koulouridis I., Alfayez M., Trikalinos T. A., Balk E. M., Jaber B. L. (2013). Dose of erythropoiesis-stimulating agents and adverse outcomes in CKD: a metaregression analysis. *American Journal of Kidney Diseases*.

[26] Kalantar-Zadeh K., Lee G. H., Miller J. E. (2009). Predictors of hyporesponsiveness to erythropoiesis-stimulating agents in hemodialysis patients. *American Journal of Kidney Diseases*.

[27] Bradbury B. D., Critchlow C. W., Weir M. R., Stewart R., Krishnan M., Hakim R. H. (2009). Impact of elevated C-reactive protein levels on erythropoiesis- stimulating agent (ESA) dose and responsiveness in hemodialysis patients. *Nephrology Dialysis Transplantation*.

[28] Adamson J. W. (2009). Hyporesponsiveness to erythropoiesis stimulating agents in chronic kidney disease: the many faces of inflammation. *Advances in Chronic Kidney Disease*.

