# Supplementary material for: Erythropoietin Dose and Mortality in Hemodialysis Patients: Marginal Structural Model to Examine Causality
Source: Int J Nephrol. 2016 May 19;2016:6087134. doi: 10.1155/2016/6087134 (PMC4889858; doi:10.1155/2016/6087134)
Supplement: Supplementary file 1 — Detailed information on cause of death was obtained from the US Renal Data System (USRDS) “CDeath” codes, which are derived from the ESRD Death Notification Form (CMS-2746) provided by ESRD networks to the USRDS. Cause of death was categorized as cardiovascular, infectious, or others by clinician decision according to these “CDeath” codes. [file 6087134.f1.pdf]

Supplement Table 1

Cause of Death
**Cardiovascular Mortality**
Myocardial Infarction, Acute
Pericarditis, Incl. Cardiac Tamponade
Atherosclerotic Heart Disease
Cardiomyopathy
Cardiac Arrest, Cause Unknown
Valvular Heart Disease
Pulmonary Edema Due To Exogenous Fluid
Congestive heart failure
Pulmonary Embolus
Cerebro-Vascular Accident Including Intracranial Hemorrhage
Ischemic Brain Damage/Anoxic Enecephalopathy
**Infectious Mortality**
Cardiac Arrhythmia
Septicemia due to internal vascular access
Septicemia due to vascular access catheter
Peritoneal access infectious complication, bacterial
Peritoneal access infectious complication, fungal
Peritonitis (complication of peritoneal dialysis)
Central nervous system infection (brain abscess, meningitis, encephalitis, etc.)
Septicemia, Due To Vascular Access *Discontinued, as of Oct 2004
Septicemia, Due To Peritonitis
Septicemia, Due To Peripheral Vascular Disease, Gangrene
Septicemia, Other
Pulmonary Infection (Bacterial)
Pulmonary Infection (Fungal))
Pulmonary Infection (Other)
Viral Infection, Cmv
Viral Infection, Other (Not 64 Or 65)
Tuberculosis
Aids
Infection, Other
Cardiac infection (endocarditis)
Pulmonary infection (pneumonia, influenza)

Abdominal infection (peritonitis-not complication of PD, perforated bowel, diverticular disease, gallbladder)
Hepatitis B
Other Viral Hepatitis
Genito-urinary infection (urinary tract infection, pyelonephritis, renal abscess)
Hepatitis C
Fungal Peritonitis
